# Craniotomy—The Child Born Alive

**Published:** 1850-03

**Authors:** 


CRANIOTOMY—THE CHILD BORN ALIVE.—A remarkable case is narrated in the annals of the Medical Society of Flanders, in which craniotomy was performed in consequence of deformed pelvis, but the child could not be extractəd. As a last resource, the Cæsarean section was performed, and, to the astonishmənt as well as horror of the surgeon, the infant was extracted alive, and exhibiting an immense lacerated wound of the skull. The brain was completely denuded, and appeared to be reduced to a complete pulp. The child survived, and suppuration was established, large quantities of brain coming away at intervals with the purulent matter. When exhibited to the Society, the child (a boy) was nine years old, and did not appear intellectually inferior to the average of boys of his age. The mother did well, and died some years afterwards of fever.—*Providence Medical and Surgical Journal, Sept.* 22.

